# Retraction Note: Protective role of p120-catenin in maintaining the integrity of adherens and tight junctions in ventilator-induced lung injury

**DOI:** 10.1186/s12931-024-02714-4

**Published:** 2024-02-07

**Authors:** Changping Gu, Mengjie Liu, Tao Zhao, Dong Wang, Yuelan Wang

**Affiliations:** Department of Anesthesiology, Qianfoshan Hospital, Shandong University, No. 16766 Jingshi Road, Jinan, 250014 Shandong Province China

The Editors-in-Chief retracted this article because of concerns regarding a number of figures presented in this work. These concerns call into question the integrity of the data and the article’s overall scientific soundness. An investigation conducted after its publication discovered instances of image overlap. The Editors-in-Chief therefore no longer have confidence in the integrity of the research presented in this article. The authors disagree to the retraction.

